# Nosocomial Isolates and Their Drug Resistant Pattern in ICU Patients at National Institute of Neurological and Allied Sciences, Nepal

**DOI:** 10.1155/2015/572163

**Published:** 2015-10-26

**Authors:** Pashupati Bhandari, Ganesh Thapa, Bharat Mani Pokhrel, Dwij Raj Bhatta, Upendra Devkota

**Affiliations:** ^1^Central Department of Microbiology, Tribhuvan University, Kathmandu 44601, Nepal; ^2^National Institute of Neurological and Allied Sciences (NINAS), Kathmandu 3711, Nepal; ^3^Department of Microbiology, Institute of Medicine (IOM), Tribhuvan University, Kathmandu 1524, Nepal

## Abstract

Multidrug resistant organisms are increasing day by day and the cause is poorly known. This study was carried out from June 2011 to May 2012 at National Institute of Neurological and Allied Sciences Kathmandu, Nepal, with a view to determining drug resistant pathogens along with detection of extended spectrum *β*-lactamase (ESBL), AmpC *β*-lactamase (ABL), and metallo-*β*-lactamase (MBL) producing bacteria causing infection to ICU patients. A standard methodology was used to achieve these objectives as per recommendation of American Society for Microbiology. ESBL was detected by combined disc assay using cefotaxime and cefotaxime clavulanic acid, ABL by inhibitor based method using cefoxitin and phenylboronic acid, and MBL by imipenem-EDTA combined disk method. Two hundred and ninety-four different clinical samples such as tracheal aspirates, urine, pus, swabs, catheter tips, and blood were processed during the study. Most common bacteria were *Acinetobacter* spp. Of the total 58 *Acinetobacter* spp., 46 (79%) were MDR, and 27% were positive for ABL and 12% were for MBL. Of the 32 cases of *Staphylococcus aureus*, 18 (56%) were MDR. Findings of this study warrant routine *β*-lactamase testing in clinical isolates.

## 1. Introduction

Intensive care unit patients are at greater risk to acquire nosocomial infection because of invasive procedures, prolonged hospital stay, high antibiotic use, cross transmission among patients and staffs, and inadequate infection control procedures which predisposes ICU as a suitable place for emergence and spread of nosocomial infections [1–3]. Most common and frequently reported nosocomial infections in ICU are urinary tract infection, ventilator associated pneumonia (VAP), surgical site infection, catheter site infection, bacteremia, and other infections like skins and soft tissue infections and common bacteria involved in such infections are* Pseudomonas aeruginosa*,* Acinetobacter* spp.,* S. aureus*,* E. coli*,* Klebsiella* spp.,* Enterobacter* spp.,* Citrobacter* spp.,* Proteus* spp., and others [4]. Sources of these organisms may be patients own flora, visitors, ICU environment like water, air, foods, and equipments, health care workers, other patients, or inanimate objects that are in close vicinity of patients [5].

Antimicrobial resistance in nosocomial infections is increasing with both morbidity and mortality especially when the infection is caused by the multidrug resistance organism [6]. More than 2 million patients are affected each year which accounts approximately for up to 10% of hospitalized patients leading to approximately 90,000 deaths per year because of nosocomial infection only [7]. Several different mechanisms for bacterial drug resistance have been described, for example, production of different drug inactivating enzymes like *β*-lactamases, multiple efflux pump, and reduced uptake [8].

This emergence and spread of antimicrobial resistance due to the production of different *β*-lactamases thus demand continual monitoring of resistance and rapid identification of such resistant organisms and determine their prevalence. Hence, this study was conducted with an aim to determine prevalence and resistance pattern of clinically relevant *β*-lactamase producers and to find antibacterial drug that could be used in therapeutics.

## 2. Methodology

This cross-sectional study was conducted from June 2011 to May 2012 at National Institute of Neurological and Allied Sciences, Kathmandu, Nepal.

### 2.1. Specimen Size and Types

294 different clinical samples, which are 152 tracheal aspirates, 43 urine samples, 31 pus/wound swabs, 24 each of CSF and CVP tips, 9 blood samples, 5 catheter tips, 2 nasal swabs, and one sample each of transsphenoidal mucosa, tissue from meningococcal cell, sputum and bone sent from ICU for routine culture, and antibiotic susceptibility tests, were processed during study period.

### 2.2. Culture

Urine specimens were cultured by semiquantitative culture technique. For urine and tracheal aspirates, a loop full of well-mixed and uncentrifuged samples was inoculated onto Blood agar (BA) and MacConkey agar (MA) and aerobically incubated at 37°C for 24 hours. CSF, pus and wound swabs were inoculated onto Blood agar (BA), MacConkey agar (MA), and Chocolate agar (CA). The BA and CA plates were incubated at 5–10% CO_2_ enriched atmosphere whereas MA was incubated aerobically at 37°C for 24 hours. Similarly tips were rolled over on the surface of the Blood agar (BA) and MacConkey agar (MA) and incubated at 37°C for 24 hours. Blood samples were first enriched on the Brain Heart Infusion broth for 48 hours and then subcultured on MA and BA every 24 hours for 3 days [9].

### 2.3. Identification of Isolates

At first colony characteristics of isolated organisms were observed on agar plates and Gram staining was performed. Gram positive isolates were further identified by using catalase, oxidase, coagulase, and optochin sensitivity tests while for identification of Gram negative isolates different biochemical tests like catalase, oxiadse, motility, H_2_S and indole production, citrate utilization, MR-VP, urea hydrolysis, and triple sugar iron utilization were done and then identified based on their results.

### 2.4. Antibiotic Susceptibility Test

Antimicrobial susceptibility of bacterial isolates was determined by Kirby-Bauer disk diffusion method as recommended by CLSI. Using sterile loop four to five different colonies of test organism were mixed with 2 mL of sterile saline and vortexed to create a smooth suspension. Turbidity of this solution is adjusted to a 0.5 McFarland standard which has corresponding bacterial concentration of approximately 150 million/mL. A sterile swab is then dipped into the suspension, firmly pressed to remove excess fluid, and plated on Muller Hinton agar. Discs were then applied on MHA plates and incubated at 37°C for 24 hours. Zone of inhibition was measured and interpreted using the standard chart and organisms reported as susceptible, intermediate, or resistant accordingly [10]. Antibiotic discs were obtained from HiMedia, Mumbai, India, and MAST Diagnostics, Merseyside, England.

### 2.5. Criterion for Multidrug Resistance

In this study, the defining criterion for an isolate to be multidrug resistant (MDR) was set as resistance to three or more drugs belonging to different structural classes [11].

### 2.6. Test for ESBL, ABL, and MBL Production

To test for ESBL production, test organism inoculum that matches McFarland tube number 0.5 turbidity was made and carpet cultured on Mueller-Hinton agar plate using sterile swab and cefotaxime (30 *μ*g) (Mast Diagnostics, UK) was applied as screening agents incubated at 37°C for 18–24 hours. Isolates showing zone of inhibition <27 mm to cefotaxime were considered as possible ESBL producers. This zone of inhibition for the cefotaxime was compared with cefotaxime (30 *μ*g) plus clavulanic acid (10 *μ*g) combination discs; an increase in zone diameter of ≥5 mm in the presence of cefotaxime plus clavulanic acid from cefotaxime alone is confirmed as ESBL producers [12].

Test organisms were screened for ABL production by using cefoxitin (30 *μ*g) disc; isolates showing zone diameters less than 18 mm were considered as screen-positive for ABL production. Screen-positive isolates were confirmed by inhibitor based method. Phenylboronic acid was prepared by dissolving 120 mg of it in 3 mL of DMSO and then 3 mL of sterile distilled water was added. Combined disc was prepared by dispensing 20 *μ*L phenylboronic acid solution to 30 *μ*g cefoxitin disc. Test was then performed by placing a disc containing 30 *μ*g cefoxitin along with a previously made combined disc containing cefoxitin and phenyl boronic acid in MHA plates by standard disc diffusion method. Plates were incubated at 37°C for 18–24 hours and zone diameter was measured. Isolates showing diameter of ≥5 mm, of zone around combined disc as compared to that of zone diameter cefoxitin disc alone, were considered as AmpC producer [13].

For MBL detection, imipenem (10 *μ*g) disc was used as a screening agent; test organisms showing intermediate or resistant zone diameter in disk diffusion method as recommended by CLSI guidelines were considered as screen-positive for MBL production. To confirm MBL detection, a 0.5 McFarland bacterial suspension was inoculated on MHA plates and two imipenem (10 *μ*g) discs were applied on the plate and in one disc 10 *μ*L of 100 mM EDTA was added directly. Plates were incubated at 37°C for 18–24 hours. Isolates showing diameter of ≥5 mm, of zone around combined imipenem-EDTA disc as compared to that of imipenem discs alone, were considered as MBL producer [14].

## 3. Results

Out of 294 total samples processed during the study, 179 (60.8%) showed significant growth with 8 polymicrobial growths. Tracheal aspirates 152 (51.7%) was the most common sample followed by urine and pus with 43 (14.6%) and 31 (10.5%) samples, respectively. Of the 152 tracheal aspirates samples, 113 (74.3%) showed significant growth among which 94 (83.1%) were MDR strains. Similarly, 25 (58.2%) and 18 (58.1%) urine and pus swab showed significant growth, respectively, among which 19 (44.1%) and 12 (66.6%) were MDR. The growth pattern and distribution of multidrug resistant isolates in different samples are presented in Table 1.

Out of 187 total isolates, 149 (79.67%) were Gram negatives and 121 (81.2%) of them were MDR.* Acinetobacter* spp. were the most frequently isolated among Gram negatives with 58 (38.9%) isolates and among them 46 (79.31%) were MDR. This was followed by* K. oxytoca* with 23 (15.4%) isolates, 20 (86.95%) of them being MDR. Similarly, out of 38 total Gram positive isolates, 21 (55.2%) were MDR and* Staphylococcus aureus* was the most common Gram positive cocci with 32 (84.2%) isolates; among them, 8 (56.25%) were MDR. The detailed results are given in Table 2.

High resistant rates of* Acinetobacter* spp. were found against antibiotics like gentamycin (70.68%), cefotaxime (82.75%), ciprofloxacin (82.75%), cefepime (86.2%), and cotrimoxazole (93.83%). Similarly, high resistance to cefotaxime and gentamycin (82.6%) each, cotrimoxazole (83.3%), ciprofloxacin and cefepime (91.3%) each, and ampicillin (100%) was found against* K. oxytoca*. Polymyxin B was found to be drug with highest sensitivity of 100% against all isolates of Gram negative rods. Detailed results are shown in Table 3.

*Staphylococcus aureus* was the major Gram positive isolate which showed higher rate of resistance to ampicillin (78.1%) while it showed sensitivity of 100% against vancomycin, followed by gentamycin (78.12%). Results are shown in Table 4.

ESBL was confirmed in 40 (32.25%) isolates; among them 10 (25%) were* E. coli* followed by 8 (20%) isolates of* K. oxytoca*. ABL was detected in 51 (31.28%) isolates and* Acinetobacter* spp. 16 (31.37%) were major ABL producers. MBL production was found in 11 isolates; among them, 7 (63.8%) were* Acinetobacter* spp. followed by 2 (18.1%) isolates each of* K. oxytoca* and* K. pneumonia*. Detailed results are presented in Table 5.

## 4. Discussion

In this study high growth rate was found from different clinical samples, and similar results have been reported in the previous study carried out at the same hospital [15, 16]. Most predominant pathogens in this study were* Acinetobacter* spp. which was in accordance with a previous study [17]. However, in other studies [18, 19] it has been shown that* Klebsiella* spp. are major nosocomial pathogens of ICU. This difference may be attributed to difference in geographical location, nutritional status, health care settings, and immune status of patient.* Acinetobacter* was also reported as the most pathogen recovered from intensive care unit patients in an international study of prevalence of “Infections in Intensive Care study” [20].

In this study, 86.9% of* K. oxytoca*, 84.21% of* Pseudomonas* spp., 81.81% of* K. pneumoniae*, and 79.31% of* Acinetobacter* spp. were multidrug resistant and similar result was also reported in an earlier study [16]. Production of different *β*-lactamases, multiple efflux pumps, decreased uptake, and other drug modifying enzymes contribute to a greater role for drug resistance in* Klebsiella* spp. and similar resistance mechanism also occurs in* Acinetobacter* spp. and* Pseudomonas* spp. [21, 22].

A higher prevalence (32.25%) of ESBL production was found in* E. coli* followed by* K. oxytoca* and* K. pneumoniae* which is in agreement with a previous study that reports a prevalence rate of 28.6%.* E. coli* and* K. pneumoniae* isolates are known to produce SHV, TEM, CTX-M, and PER types of ESBLs and show variable resistance to *β*-lactam antibiotics resulting in therapeutic failure [23]. Several risk factors exist for colonization and infection with ESBL producer like seriously ill patients with prolonged hospital stay, use of invasive devices, heavy and prior antibiotic use, poor nutritional status, recent surgery, gastrostomy, total parenteral nutrition, and hemodialysis [24].

High prevalence of AmpC *β*-lactamase was detected in* Acinetobacter* spp. (29.4%) followed by* Staphylococcus aureus* (21.5%) and* K. oxytoca* (15.6%) which follows pattern in accordance with the previous result [25] with a prevalence rate of 20% in* Klebsiella* spp. High level of AmpC production is typically associated with the resistance to all *β*-lactam antibiotics except carbapenems and limits the therapeutic use. Sensitivity and specificity of the method used in this study are 90% and 98.2%, respectively [13].

In 11 isolates MBL was detected out of 17 screen-positive isolates with prevalence rate of 64.7%; among them 7 (63.63%) were* Acinetobacter* spp. and the rest* Klebsiella* spp. Different transferable MBL is found in these organisms and major ones are IMP, VIM, and SIM type [26]. Contrary to current finding a Korean survey showed only 6% MBL positive isolates [27]. The increasing trend of carbapenem resistance in* Acinetobacter* spp. worldwide poses a significant concern since it limits the range of therapeutic alternative. Carbapenem resistance in* Acinetobacter* is due to naturally occurring *β*-lactamases, acquired *β*-lactamases like metallo-*β*-lactamases, carbapenem hydrolyzing oxacillinases (CHDLs), loss of outer membrane porin protein, and sometimes modification in penicillin-binding protein [28].

## 5. Conclusion

*Acinetobacter* spp. and* S. aureus* were major pathogens prevalent in ICU of National Institute of Neurological and Allied Sciences during the study. Inclusion of ESBL, ABL, and MBL in clinical isolates is warranted.

## Figures and Tables

**Table 1:** Growth pattern and distribution of MDR isolates in different samples.

Specimen	Number of samples	Growth number (%)	Number (%) of MDR strains
Tracheal aspirates	152	113 (74.3)	94 (83.1)
Urine	43	25 (58.2)	19 (44.1)
Pus/wound swab	31	18 (58.1)	12 (66.6)
CVP tip	24	10 (41.6)	10 (100)
CSF	24	4 (17.3)	3 (75)
Blood	9	1 (11.1)	0
ICP catheter	3	1 (25)	1 (33.3)
EVD drain tip	2	2 (100)	2 (100)
Nasal swab	2	2 (100)	0
Others	4	3 (75)	1 (33.3)

Total	294	179 (60.88)	142 (79.3)

Others include tissue from meningococcal cell, transsphenoidal mucosa, bone, and sputum.

**Table 2:** Frequency of bacterial isolates and their multidrug resistant profile.

SN	Bacterial isolates	Total isolate number	Multidrug resistance isolates number (%)
1	*Acinetobacter* spp.	58	46 (79.31)
2	*K. oxytoca*	23	20 (86.95)
3	*K. pneumoniae *	22	18 (81.81)
4	*E. coli*	19	14 (73.62)
5	*Pseudomonas *spp.	19	16 (84.21)
6	*Citrobacter *spp.	3	2 (66.66)
7	*P. vulgaris*	3	3 (100)
8	*P. mirabilis*	2	2 (100)
9	*Staphylococcus aureus*	32	18 (56.25)
10	*β*-hemolytic streptococci	3	3 (100)
11	Viridans streptococci	2	0
12	Coagulase negative staphylococci	1	0

Total		187	142 (75.93)

Multidrug resistance criteria: resistance to three or more drugs of different structural classes.

**Table 3:** Antibiotics profile of major Gram negative pathogens.

Antibiotics	*Acinetobacter *spp. (*n* = 58)	*K. oxytoca* (*n* = 23)	*K. pneumoniae* (*n* = 22)	*Pseudomonas *spp. (*n* = 19)	*E. coli * (*n* = 19)
Ampicillin	NT	100	100	NT	78.11
Amikacin	67.24	73.91	59.09	31.57	19.04
Cotrimoxazole	93.83	83.31	81.8	84.21	68.85
Cefotaxime	82.75	82.6	81.0	73.68	78.94
Cefepime	86.20	91.3	81.81	84.4	57.8
Carbenicillin	NT	NT	NT	42.1	NT
Ciprofloxacin	82.75	91.3	68.18	73.68	73.68
Gentamycin	70.68	82.6	59.09	42.1	47.36
Imipenem	17.24	0	18.18	0	0
Ofloxacin	68.96	60.80	63.63	47.36	63.15
Piperacillin/tazobactam	50.02	40.90	63.15	5.26	15.78
Polymyxin B	0	0	0	0	0

NT: not tested.

**Table 4:** Antibiotic susceptibility profile of *S. aureus *(*n* = 32).

Antibiotic used	Sensitive		Resistant	
	Number	%	Number	%
Ampicillin	7	21.9	25	78.1
Cotrimoxazole	18	56.25	14	43.7
Cefotaxime	19	59.38	13	40.62
Cefoxitin	18	56.25	14	43.75
Ciprofloxacin	23	71.87	9	28.12
Cloxacillin	20	62.5	12	37.5
Gentamycin	25	78.12	7	21.87
Methicillin	22	68.75	10	31.25
Ofloxacin	24	75	8	25
Vancomycin	100	100	0	0

**Table 5:** ESBL versus ABL versus MBL producing bacteria.

Bacteria	ESBL production number (%)	ABL production number (%)	MBL production number (%)
*E. coli*	10 (25%)	4 (7.8%)	0
*K. oxytoca*	8 (20%)	8 (15.6%)	2 (18.1%)
*K. pneumoniae*	6 (15%)	6 (11.7%)	2 (18.1%)
*Acinetobacter *spp.	5 (12.5%)	16 (31.37%)	7 (63.8%)
*Pseudomonas *spp.	5 (12.5%)	4 (7.8%)	0
*Citrobacter *spp.	1 (2.5%)	2 (3.9%)	0
*P. mirabilis*	1 (2.5%)	0	0
*P. vulgaris*	1 (2.5%)	0	0
*S. aureus*	3 (7.5%)	11 (21.5%)	0

Total	40 (32.25%)	51 (31.28%)	11 (64.7%)
